# Commonalities and differences in trait-like, risky, and utilitarian decision-making styles between abstinent heroin-dependent individuals and their siblings

**DOI:** 10.3389/fpsyt.2025.1659008

**Published:** 2026-01-08

**Authors:** Wan-Sen Yan, Yan Lan, Su-Jiao Liu

**Affiliations:** 1 Department of Psychology, School of Medical Humanitarians, Guizhou Medical University, Guiyang, China; 2 Guizhou Research Institute for Health Development, Guizhou Medical University, Guiyang, China

**Keywords:** decision-making, heroin dependence, risk-taking, siblings, utilitarian choice

## Abstract

**Background:**

Heroin dependence is associated with poor performance on laboratory-based decision-making paradigms. However, it remains unclear whether these deficits may have predated drug abuse due to potential familial susceptibilities or emerged as a consequence of chronic drug use. A family study may help clarify this important issue, so this study was to compare various decision-making aspects between heroin-dependent individuals and their siblings.

**Methods:**

A total of 70 abstinent heroin-dependent individuals (HAs), 69 unaffected biological siblings of the HAs (Siblings), and 74 unrelated healthy subjects (HCs) were included and tested on trait-like, risky, and utilitarian decision-making domains, using the Melbourne Decision-Making Questionnaire (MDMQ), the Balloon Analogue Risk Task (BART), and the Moral Decision-Making Task (MDMT).

**Results:**

Data indicated that both HAs and Siblings scored higher on MDMQ Procrastination (Cohen’s *d* = 0.61-1.17), and exhibited higher risk-taking levels (i.e., more pumps per trial and more explosions) on the BART (Cohen’s *d* = 0.39-1.25) compared with HCs. A lower level of competent decision-making (MDMQ Vigilance) and a higher ratio of utilitarian choices in self-involvement dilemmas were found in HAs, but not in Siblings, compared to HCs. Logistic regression models revealed homologous results.

**Conclusions:**

These findings suggest that deficits in trait-like and risk-taking-related decision-making styles are shared by abstinent heroin-dependent individuals and their unaffected siblings, which might represent the conceivable markers for potential familial vulnerabilities implicated in the development of heroin dependence.

## 1 Introduction

Heroin abuse and dependence have traditionally been significant public health and social issues. In recent years, the new wave of heroin and other opioids (such as synthetic fentanyl) epidemic has once again captured the attention of scientists worldwide (1–3). Individuals with chronic heroin use are at a high risk for various devastating consequences, particularly fatalities due to overdose (4, 5). However, the neurobiological mechanisms underlying heroin dependence remain elusive (6). Chronic heroin use is associated with several neuropsychological impairments, including deficits in cognitive flexibility, inhibitory control, working memory, planning, and decision-making, even after a period of abstinence (7–12). In daily life, heroin abusers tend to seek and consume drugs instead of seeking treatment and overcoming the habits of drug abuse. Actually, repeated use of addictive drugs in the face of negative consequences implies a dysfunctional mechanism underlying decision making in these individuals (13).

Theories of addiction that address cognitive-dysfunction views of drug use (impaired prefrontal top-down control), such as the competing-neurobehavioral-decision-systems (CNDS) model (1), have proposed three key neural systems involved in decision-making processes implicated in drug addiction, namely, an amygdala-striatum-based impulsive system, a prefrontal cortex-based executive system, and an insula- based modulating system (1, 14, 15). It is believed that abnormal functioning in one or more of these three systems may all act together to influence addiction (1). In laboratory-based decision- making tasks, numerous studies have identified significant impairments among individuals with heroin abuse and dependence (16–19), consistent with the CNDS model and empirical observations.

Specifically, when assessed with neuropsychological tasks such as the Iowa Gambling Task (IGT) and Cambridge Gambling Task (CGT), heroin abusers exhibited compromised decision-making abilities, preferring immediate profitable but long-term disadvantageous choices (8, 11, 20–23). In risky decision-making tasks that evaluate risk-taking propensity, such as the Game of Dice Task (GDT) and Balloon Analogue Risk Task (BART), individuals with heroin dependence showed an increased inclination towards risk-taking in pursuit of the rewards (24–26). Furthermore, heroin abusers revealed a higher level of impulsive decision-making than healthy subjects on the Delay Discounting Task (DDT) (8, 21, 27–29). Despite a few contradictory studies, these findings imply that decision-making appears to be one of the neurocognitive functions most consistently and severely affected in heroin abuse and dependence (16). More importantly, decision-making deficits in heroin-dependent individuals have not been shaped by the length of abstinence, suggesting that recovery in these functions is minimal at best (8, 17). Compromised decision-making ability in heroin abuse is further supported by neuroimaging studies that indicated long-lasting abnormalities in the orbitofrontal cortex, medial prefrontal cortex, and associated neural networks linked to decision-making processes in chronic heroin abusers (9, 30).

However, apart from the aforementioned domains of decision-making, the existing evidence for the other aspects of decision-making is less clear in heroin use disorder, such as self-reported trait-like decision-making styles (31) and moral decision-making (32–34). Like typical personality traits that have been considered as potential vulnerability markers for drug addiction (e.g., impulsivity) (35), decision-making styles at the trait level may play an important role in the vulnerability to addiction (36). In one earlier study, the Melbourne Decision-Making Questionnaire (MDMQ) (37) was employed to assess self-reported decision-making styles in drug-dependent individuals (31), finding that stimulant abusers showed less competent (vigilance) and more maladaptive (procrastination) styles, while opiate abusers performed similarly to healthy subjects. As for moral decision-making, moral dilemmas are utilized to contrast utilitarian choices (based on consequences) and deontological choices (based on moral norms) traditionally (38). Patients with alcohol use disorders (39) and polysubstance dependence (32, 33) presented a utilitarian bias (i.e., made more utilitarian choices) in moral decision-making tasks compared to healthy subjects. As a special instance of decision-making, moral judgment relies on the integration of cognitive and emotional signals in order for individuals to determine how to resolve moral dilemmas effectively (38). Accordingly, impaired cognitive functioning and/or reduced empathy may lead to a utilitarian bias in moral judgment (40), which is supported by functional abnormalities in fronto-limbic systems (e.g., anterior cingulate, insula, amygdala) observed in cocaine and stimulant abusers when completing moral decision-making tasks (34, 41). Nevertheless, the profiles of trait-like and moral decision-making in heroin use disorder remain ill-defined, given the scarcity of evidence.

Furthermore, one intriguing topic has been raised that whether decision-making impairments may preexist or be induced (exacerbated) by chronic heroin abuse (1, 42). In this respect, family design studies are of great help to parse genetic factors and sequalae of drug use (26). Previous research about stimulant abuse has compared those addicted to stimulants and their unaffected biological siblings on neurocognitive tasks, indicating that inhibitory control is a neurocognitive endophenotype in stimulant use disorder (43– 45). In heroin use disorder, a recent study revealed deficits in cognitive flexibility and inhibitory control both in heroin-dependent patients and their unaffected biological siblings (12). It is thus plausible that cognitive dysfunctions might predate the initiation of drug use, rendering individuals more predisposed to developing an addiction if certain conditions are met, despite that chronic drug use can also cause and aggravate neurocognitive impairments due to neurotoxicity (1). More interestingly, assessed with decision-making tasks, sibling pairs discordant for heroin dependence were significantly correlated on delay aversion and risk-taking propensity (26). Additionally, longitudinal studies have found that worse decision-making capacities can predict the course of subsequent substance use and non-substance-related addictive disorders (46, 47). These fascinating yet limited findings, necessitate further studies to examine whether decision-making reflects a vulnerability to heroin use disorder.

Considering the above-mentioned topics, we adopted a family study design in the current research, directly contrasting abstinent heroin-dependent individuals and their unaffected biological siblings with well-matched healthy subjects. Trait-like, moral, and risky decision-making domains were tested using the Melbourne Decision-Making Questionnaire (MDMQ), Moral Decision-Making Task (MDMT), and Balloon Analogue Risk Task (BART). We hypothesized that both the heroin-dependent individuals and their unaffected siblings would show deficits in these domains as potential familial vulnerabilities.

## 2 Methods

### 2.1 Participants and procedure

Participants included 70 abstinent heroin-dependent individuals (HAs), 69 unaffected healthy biological siblings of the HAs (Siblings), and 74 unrelated healthy subjects (HCs). This sample has been reported in detail elsewhere (12). Briefly, all subjects were firstly evaluated using the Structured Clinical Interview for DSM-IV disorders (48) by an experienced psychiatrist and a clinical psychologist, and then finished a battery of self-report scales and behavioral tasks in the laboratory. The HAs (mean age: 43.23 ± 7.62 years, ranging from 27 to 58 years; 49 males, 70%) were recruited at a local hospital rehabilitation center for drug addiction at Guiyang, China. They were voluntarily enrolled in this study. All of them met the diagnostic criteria for a lifetime history of heroin dependence, and were “pure” heroin users, with those who had abused more than one kind of addictive drugs in addition to heroin (e.g., cocaine, methamphetamine, ketamine) excluded from the enrollment. The average duration of heroin use for the HAs was 15.20 ± 5.77 years (from 1 to 24 years), and average abstinence from last drug use was 12.86 ± 3.80 months (from 3 to 18 months). Exclusion criteria for the HAs included: (1) less than 18 or ≥ 60 years of age; (2) a personal history of alcohol dependence, psychiatric disorders (e.g., psychotic episodes, schizophrenia, major depressive disorder) or a history of brain injury/trauma; (3) current/past neurological diseases or mental disorders, or severe physical conditions. Sixty-nine unaffected healthy biological siblings of the HAs were enrolled. Siblings were included when they met: (1) having same biological parents with the other sibling in the HAs; (2) aged 18-59 years; (3) having no personal history of alcohol and drug abuse or dependence. Exclusion criteria for the Siblings included: (1) a personal history of psychiatric disorders (e.g., psychotic episodes, schizophrenia, major depressive disorder) or brain injury/trauma; (2) current/past neurological diseases or mental disorders, severe medical illness or physical conditions. The 74 HCs were recruited in local community, matched on sex, age, and ethnicity with the HAs and the Siblings. Inclusion criteria for the HCs included: (1) aged 18-59 years; (2) having no personal and family history of alcohol and drug abuse or dependence. Exclusion criteria for the HCs were identical to those for the Siblings.

### 2.2 Ethics

All participants provided written informed consent, and were compensated with RMB ¥50 each for their time. This study was approved by the Human Research Ethics Committee at Guizhou Medical University (GMU/2020LS05), and performed in line with the principles of the Declaration of Helsinki.

### 2.3 Measures

#### 2.3.1 Decision-making tasks

##### 2.3.1.1 Trait-like decision-making

We used the Melbourne Decision-Making Questionnaire (MDMQ) to assess self-reported decision-making styles at the level of personality traits. The MDMQ is a 22-item self-report instrument for measuring coping patterns in decision-making situations (37). It consists of four dimensions, including Vigilance (6 items), Buck-passing (6 items), Procrastination (5 items), and Hypervigilance (5 items). Each is calculated with a sum score separately. Vigilance is a competent decision-making style, characterized by a rational, unbiased search for relevant information and the careful appraisal of all alternatives prior to decision making. Buck-passing (i.e., deferring decisions to others), Procrastination (i.e., delaying decisions), and Hypervigilance (i.e., quickly making a choice to escape the pressure) are maladaptive styles that usually lead to suboptimal outcomes. The Cronbach’s α for these four subscales in this study was 0.745, 0.723, 0.687, and 0.707, respectively.

##### 2.3.1.2 Risky decision-making

The Balloon Analogue Risk Task (BART) was used to assess individuals’ propensity for risk-taking in the context of risky decision-making (49). The BART allows subjects to earn a small monetary reward for each pump to blow up a balloon, but at some point, the balloon explodes. In this task, participants are asked to pump the balloon, and if they choose to cash out before the balloon explodes, they keep the money; however, if the balloon explodes, they will lose the money earned during that trial. The main outcome indices for the BART include an average number of pumps on unexploded balloons (i.e., adjusted average pumps) and the number of exploded balloons (49, 50), with higher scores reflecting greater risk-taking propensity.

##### 2.3.1.3 Moral decision-making

The Moral Decision-Making Task (MDMT) was used to assess utilitarian moral judgment in a set of trolley-like driving dilemmas (51). In the MDMT, there are 20 incidental scenarios of driving-type dilemmas (i.e., killing one person as a foreseen but unintended consequence of saving many lives). Each of the driving-type dilemmas consists of a hypothetical moral text scenario and two possible resolutions (a deontological action *vs.* a utilitarian action). In this task, self-risk involvement factors are considered. In 10 scenarios, subjects are not involved as a potential victim of the accident (i.e., other-involvement dilemmas), while in the other 10 scenarios, the utilitarian outcomes result in protecting one’s own and other people’s lives, but sacrificing a single individual as an unintended and predicted side effect (i.e., self-involvement dilemmas). One example is as follows: *Scenario:* “*It is night, and you are driving your car. A violent storm has hit your city for a few hours; it is still raining, and the asphalt is slippery. You are approaching a traffic light when suddenly two cyclists cross the road right in front of you. On the right sidewalk, you see a pedestrian. You try to brake, but the tires slide on the asphalt.*” *Resolutions: (A) You continue straight, running over the two cyclists, who will die. Your car will continue to slide on the asphalt, crashing against a nearby building, and you will die; (B) You suddenly steer right. You know you will run over the pedestrian on the sidewalk, who will die, but your car will slow down in an open field and you, and the two cyclists will be unhurt*. In this scenario (self-involvement dilemma), the resolution A is considered a deontological action, while the resolution B is deemed as a utilitarian action. As the primary outcome indicator for the MDMT, we calculated the ratio of utilitarian choices made by each subject, separately in the scenarios involving self-involvement and other-involvement dilemmas, as suggested before (51).

#### 2.3.2 Other self-report scales

The Self-rating Anxiety Scale (SAS) (52) and Self-rating Depression Scale (SDS) (53) were used to measure anxiety and depression symptoms over the past two weeks. Both scales are rated on a 4-point Likert scale (1 = none or a little of the time, 4=most or all of the time). Higher scores reflect heavier symptoms. Cronbach’s α for these two scales was 0.79 and 0.80 in this study, respectively.

General intelligence were evaluated by using the Raven’s Standard Progressive Matrices (54, 55), a 60-item standard non-verbal test for measuring abstract reasoning (fluid intelligence). All participants also filled in a brief questionnaire for demographic data (e.g., age, gender, ethnicity, education years, smoking status, drinking status, and drug use variables).

### 2.4 Statistical analyses

Data were analyzed with the Statistical Package for the Social Sciences for Windows, Version 22.0 (SPSS Inc., Chicago, IL, USA). Demographic variables were evaluated and compared across groups. Chi-Square tests were conducted between groups on gender, ethnicity, drinking status, and smoking status (proportion of current smokers). One-way analysis of variance (ANOVA) were tested between groups on age, years of education, general intelligence, as well as SAS and SDS scores. Due to the significant between-group differences on smoking status and SAS and SDS scores, the multivariate analysis of covariance (mACOVA) models with a 3 (Group: HAs, Siblings, HCs) × 2 (Smoking status: current smokers, non-smokers) design were adopted on main task scores (i.e., MDMQ, BART, MDMT), with SAS and SDS scores entered as two covariates. In order to test the independence of the dependent variables (MDMQ, BART, and MDMT scores) in the mACOVA models, we calculated the intraclass correlation coefficient (ICC) for these variables with a two-way mixed effect model. The ICC was small to moderate (single measure: ICC = 0.071, 95% CI = 0.039-0.110; average measure: ICC = 0.378, 95% CI = 0.243-0.497). *Post-hoc* tests were conducted using Fisher’s least significant differences (LSD) protected *t*-test. The relationships of main task scores (i.e., MDMQ, BART, MDMT) with SAS and SDS scores, and the associations between drug use variables and main task scores in the HAs were tested with Pearson correlations. In addition, three logistic regression models were used to test the discriminant effects of main task scores on the groups, each for HAs *vs.* HCs, Siblings *vs.* HCs, and HAs *vs.* Siblings, respectively. Multicollinearity was not a problem based on the variance inflation factor (VIF<5) in the regression models. Statistical significance was set as *p* < 0.05, two-tailed.

## 3 Results

### 3.1 Demographic characteristics

No significant differences were displayed on gender, age, ethnicity, years of education, general intelligence, or drinking status between the three groups (**Table 1**). Group difference on smoking status was significant such that the proportion of current smokers was much higher in HAs (78.6%) than that in Siblings (49.3%) and HCs (29.7%). *Post-hoc* tests revealed that Siblings also had a higher proportion of current smokers than HCs (*p* < 0.05). Moreover, group differences were significant both for SAS (*F*_(2, 210)_=5.325, *p=*0.006) and SDS (*F*_(2, 210)_=10.561, *p* < 0.001). *Post-hoc* tests showed that HAs scored higher than Siblings and HCs (*ps* < 0.01), but Siblings did not differ with HCs on the SAS and SDS (*ps*>0.05).

**Table 1:** Demographic data of the three groups.

Variables	HAs (*n* = 70)	Siblings (*n* = 69)	HCs (*n* = 74)	*χ²/F*	*p*
Gender, male *n* (%)	49 (70.00)	50 (72.46)	52 (70.27)	0.123	0.940
Age, years (*M ± SD*)	43.23 ± 7.62	42.61 ± 8.37	41.15 ± 8.38	1.247	0.290
Years of education (*M ± SD*)	13.21 ± 2.93	13.61 ± 2.68	14.14 ± 2.84	1.935	0.147
General intelligence (*M ± SD*) ^a^	104.60 ± 7.79	105.03 ± 7.40	107.04 ± 7.98	2.058	0.130
Ethnicity, Hans *n* (%)	62 (88.57)	61 (88.41)	64 (86.49)	0.182	0.913
Drinking status, *n* (%) ^b^				4.749	0.314
Never	35 (50.00)	45 (65.22)	48 (64.87)		
1 or 2 days	31 (44.29)	22 (31.88)	24 (32.43)		
3-9 days	4 (5.71)	2 (2.90)	2 (2.70)		
≥ 10 days	0 (0.00)	0 (0.00)	0 (0.00)		
Smoking status					
Current smokers, *n* (%)	55 (78.57)	34 (49.28)	22 (29.73)	34.715	< 0.001
Drug use variables (*M ± SD*)					
Years of heroin use	15.20 ± 5.77	NA	NA		
Age at first heroin use	26.13 ± 6.29	NA	NA		
Months from last use	12.86 ± 3.80	NA	NA		
SAS scores (*M ± SD*)	31.51 ± 7.25	29.19 ± 4.69	28.53 ± 4.94	5.325	0.006
SDS scores (*M ± SD*)	36.50 ± 5.92	33.22 ± 5.10	32.74 ± 4.84	10.561	< 0.001

a Estimated by the intelligence quotient (IQ) scores on the Raven’s Standard Progressive Matrices (RSPM).

b Rated on the single item: How many days have you had at least one drink of alcohol during the past 30 days?

SAS, the Self-Rating Anxiety Scale. SDS, the Self-rating Depression Scale. NA, Not Applicable.

HAs, Heroin-dependent individuals; HCs, Healthy subjects.

### 3.2 Group differences on task scores

The mean scores on the MDMQ, BART, and MDMT for the three groups are shown in **Table 2**.

**Table 2:** Task scores on the MDMQ, BART, and MDMT for the three groups (*M ± SD*).

Variables	HAs (*n* = 70)	Siblings (*n* = 69)	HCs (*n* = 74)	*F*	*p*
MDMQ					
Vigilance	6.96 ± 3.15	8.90 ± 1.64	9.05 ± 1.89	18.012	< 0.001
Buck-passing	4.04 ± 3.16	3.91 ± 2.12	3.30 ± 2.83	1.528	0.219
Procrastination	5.54 ± 2.20	4.25 ± 1.61	3.24 ± 1.73	27.457	< 0.001
Hypervigilance	3.17 ± 2.65	3.20 ± 2.03	2.95 ± 2.35	0.255	0.775
BART					
Adjusted average number of pumps	22.19 ± 6.93	18.74 ± 7.45	15.93 ± 6.21	14.952	< 0.001
Total number of explosions	11.85 ± 3.82	8.56 ± 5.79	6.53 ± 4.63	22.348	< 0.001
MDMT					
Ratio of utilitarian choices (self-involved scenarios), %	87.68 ± 16.55	78.80 ± 17.05	76.69 ± 17.96	8.155	< 0.001
Ratio of utilitarian choices (other-involved scenarios), %	70.54 ± 21.71	66.67 ± 26.23	67.57 ± 24.56	0.488	0.615

MDMQ, the Melbourne Decision-Making Questionnaire. BART, the Balloon Analogue Risk Task.

MDMT, the Moral Decision-Making Task. HAs, Heroin-dependent individuals; HCs, Healthy subjects.

On the MDMQ, the mACOVA models revealed significant main effects of group on Vigilance (*F*_(2, 205)_ =22.184, *p* < 0.001, *η*_p_^2^ = 0.178) and Procrastination (*F*_(2, 205)_ =10.260, *p* < 0.001, *η*_p_^2^ = 0.091), but not on Buck-passing (*F*_(2, 205)_=0.116, *p* = 0.891) or Hypervigilance (*F*_(2, 205)_=0.300, *p* = 0.741). *Post-hoc* tests indicated that HAs scored lower than both Siblings and HCs (*ps* < 0.001), but Siblings were comparable with HCs on Vigilance (*p* = 0.292); while on Procrastination, both HAs and Siblings scored higher than HCs (Cohen’s *d* = 1.17, *p* < 0.001; Cohen’s *d* = 0.61, *p* = 0.016, respectively), and HAs scored higher than Siblings (Cohen’s *d* = 0.67, *p* = 0.012). Effects of smoking status and group × smoking status were not significant on any scores (*F*_(1, 205)_=0.660-3.042, *ps*>0.05; *F*_(2, 205)_=0.751-2.577, *ps*>0.05, respectively).

On the BART, significant main effects of group were displayed both on average number of pumps (*F*_(2, 205)_ =8.743, *p* < 0.001, *η*_p_^2^ = 0.079) and number of explosions (*F*_(2, 205)_ =13.330, *p* < 0.001, *η*_p_^2^ = 0.115). Effects of smoking status and group × smoking status were not significant on any task scores (*F*_(1, 205)_=0.071-0.446, *ps*>0.05; *F*_(2, 205)_=0.972-1.256, *ps*>0.05, respectively). *Post-hoc* tests indicated that both HAs and Siblings exhibited more pumps per trial on unexploded balloons than HCs (Cohen’s *d* = 0.95, *p* < 0.001; Cohen’s *d* = 0.41, *p* = 0.034, respectively), and HAs performed more pumps on average than Siblings (Cohen’s *d* = 0.48, *p* = 0.015). Moreover, both HAs and Siblings showed more exploded balloons during the task than HCs (Cohen’s *d* = 1.25, *p* < 0.001; Cohen’s *d* = 0.39, *p* = 0.040, respectively), and HAs had more total explosions than their Siblings on the BART (Cohen’s *d* = 0.67, *p=*0.001).

On the MDMT, the mACOVA models revealed significant main effects of group on the ratio of utilitarian choices in the self-involvement scenarios (*F*_(2, 205)_ =7.345, *p* = 0.001, *η*_p_^2^ = 0.067), but not in the other-involvement scenarios (*F*_(2, 205)_ =0.818, *p* = 0.443). Effects of smoking status and group × smoking status were not significant (*F*_(1, 205)_=0.237-0.648, *ps*>0.05; *F*_(2, 205)_=0.399-1.093, *ps*>0.05, respectively). *Post-hoc* tests indicated that HAs made a higher proportion of utilitarian decisions than both Siblings and HCs in self-involvement dilemmas (*ps* < 0.001), but Siblings were comparable with HCs (*p* = 0.669).

### 3.3 Correlations between heroin use variables and task scores

In the HAs, years of heroin use were not significantly related to any MDMQ scores (*r*_(70)_ = 0.019-0.184, *ps*>0.05), BART number of explosions (*r*_(70)_ = 0.173, *p=*0.153), or MDMT scores (*r*_(70)_ = 0.001-0.016, *ps*>0.05), but were positively related to BART average number of pumps (*r*_(70)_ = 0.281, *p=*0.018). Similarly, age of first use was significantly related to BART average number of pumps (*r*_(70)_ = -0.294, *p=*0.014), but not related to any other scores (*ps*>0.05). Months of abstinence were also not significantly related to any of the MDMQ, BART, and MDMT scores (*r*_(70)_ = 0.017-0.210, *ps*>0.05).

In all subjects, the MDMQ, BART, and MDMT scores were not significantly related to the SAS and SDS scores (*r* = 0.004-0.132, *ps*>0.05) or general intelligence (*r* = 0.002-0.139, *ps*>0.05), please see the **Supplementary Materials** for more details about the correlations.

### 3.4 Logistic regression outcomes

We conducted three logistic regression models with a two-step design. Firstly, smoking status, SAS scores, and SDS scores were entered as control variables at Step 1, considering their significant between-group differences. At Step 2, MDMQ, BART, and MDMT scores were entered as predictive variables. Group was the dependent variable in these models (i.e., Model 1 for HAs *vs.* HCs, Model 2 for Siblings *vs.* HCs, and Model 3 for HAs *vs.* Siblings). As shown in **Table 3**, in Model 1 (*Nagelkerke R^2^ * = 0.421) and Model 2 (*Nagelkerke R^2^ * = 0.225), MDMQ Procrastination and BART average number of pumps are two shared predictors that are positively linked to both HAs and Siblings compared with HCs (*OR* = 1.467-2.095, *ps* < 0.01; *OR* = 1.069-1.218, *ps* < 0.05, respectively). By contrast, lower MDMQ Vigilance and more BART explosions are two specific factors that are associated with HAs compared to HCs (*OR* = 0.537, *p* < 0.001; *OR* = 1.146, *p* < 0.05, respectively). In **Table 4**, the Model 3 (*Nagelkerke R^2^ * = 0.248) shows that lower MDMQ Vigilance, higher MDMQ Procrastination, and more BART explosions are connected with HAs compared to Siblings (*OR* = 0.546, *p* < 0.001; *OR* = 1.601, *p* < 0.01; *OR* = 1.115, *p* < 0.05, respectively).

**Table 3:** Logistic regression models for the task scores on predicting HAs and Siblings *vs* HCs.

Models	Model 1: HAs (1) *vs.* HCs (0), *n* = 144			Model 2: Siblings (1) *vs.* HCs (0), *n* = 143		
	B	Wald *χ²*	OR (95% CI)	B	Wald *χ²*	OR (95% CI)
Step 1						
Current smokers (yes=1, no=0)	2.542	12.745***	12.706 (3.147-51.296)	0.826	4.270*	2.283 (1.043-4.997)
SAS scores	0.104	2.805	1.110 (0.982-1.254)	0.042	1.092	1.043 (0.964-1.129)
SDS scores	0.089	1.895	1.093 (0.963-1.240)	0.021	0.275	1.021 (0.944-1.105)
Step 2						
MDMQ Vigilance	-0.622	12.056***	0.537 (0.378-0.763)	-0.036	0.110	0.964 (0.779-1.194)
MDMQ Buck-passing	0.305	2.837	1.263 (0.517-2.051)	-0.042	0.172	0.959 (0.788-1.168)
MDMQ Procrastination	0.740	9.390**	2.095 (1.305-3.362)	0.383	6.753**	1.467 (1.099-1.958)
MDMQ Hypervigilance	0.149	0.487	1.160 (0.764-1.761)	-0.089	0.641	0.915 (0.737-1.137)
BART average number of pumps	0.197	5.467*	1.218 (1.032-1.438)	0.067	5.598*	1.069 (1.012-1.130)
BART total number of explosions	0.145	4.975*	1.146 (1.016-1.689)	0.047	1.467	1.048 (0.971-1.132)
MDMT ratio of utilitarian choices ^a^	0.014	0.045	1.014 (0.894-1.149)	0.013	1.163	1.013 (0.989-1.037)
MDMT ratio of utilitarian choices ^b^	0.019	1.355	1.019 (0.987--1.051)	-0.009	0.992	0.991 (0.974-1.009)

^a^Self-involved scenarios. ^b^Other-involved scenarios. HAs, Heroin-dependent individuals; HCs, Healthy subjects.

SAS, the Self-Rating Anxiety Scale. SDS, the Self-rating Depression Scale.

MDMQ, the Melbourne Decision-Making Questionnaire. BART, the Balloon Analogue Risk Task.

MDMT, the Moral Decision-Making Task. *p < 0.05, **p < 0.01, ***p < 0.001.

**Table 4:** Logistic regression models for the task scores on predicting HAs *vs* siblings.

Models	Model 3: HAs (1) *vs.* siblings (0), *n* = 139		
	B	Wald *χ²*	OR (95% CI)
Step 1			
Current smokers (yes=1, no=0)	1.802	9.758**	6.061 (1.957-18.776)
SAS scores	0.115	6.657*	1.122 (1.028-1.225)
SDS scores	0.068	2.085	1.071 (0.976-1.174)
Step 2			
MDMQ Vigilance	-0.604	18.153***	0.546 (0.414-0.722)
MDMQ Buck-passing	-0.230	3.001	0.794 (0.612-1.031)
MDMQ Procrastination	0.471	7.962**	1.601 (1.155-2.221)
MDMQ Hypervigilance	0.179	1.365	1.196 (0.886-1.616)
BART average number of pumps	0.034	0.712	1.034 (0.956-1.119)
BART total number of explosions	0.109	4.305*	1.115 (1.006-1.237)
MDMT ratio of utilitarian choices ^a^	0.012	1.157	1.012 (0.987-1.039)
MDMT ratio of utilitarian choices ^b^	-0.007	0.286	0.993 (0.970-1.018)

^a^Self-involved scenarios. ^b^Other-involved scenarios. HAs, Heroin-dependent individuals; HCs, Healthy subjects.

SAS, the Self-Rating Anxiety Scale. SDS, the Self-rating Depression Scale.

MDMQ, the Melbourne Decision-Making Questionnaire. BART, the Balloon Analogue Risk Task.

MDMT, the Moral Decision-Making Task. *p < 0.05, **p < 0.01, ***p < 0.001.

## 4 Discussion

In this study, we directly investigated trait-like, risky, and utilitarian decision-making domains in abstinent heroin-dependent individuals and their unaffected biological siblings compared to unrelated healthy subjects. Convergent data from the mACOVA and logistic regression models revealed that specific trait-like decision-making styles (Procrastination) and risk-taking propensity were elevated in both HAs and Siblings, representing conceivable markers associated with familial vulnerabilities in heroin use disorder. However, lower competent decision-making abilities (Vigilance) and a utilitarian bias were merely found in HAs, which might reflect the negative consequences of chronic heroin use.

Decision-making is a high-order cognitive process and plays an important role in the survival and development of human beings. Patients with brain damage often make disadvantageous decisions on the IGT that simulates real-life decision-making (56–58). Individuals with drug addiction also exhibit deficits in decision-making, characterized by a preference for immediate rewards but a disregard for the long-term consequences (59). Particularly, both current and former opiate abusers showed robust decision-making impairments, which were not significantly moderated by comorbid factors and were unrelated to the length of abstinence (17). The current literature suggests that decision-making appears to be one of the most severely affected neurocognitive functions in addiction, which is consistently found in heroin use disorder (16). In this study, we focused on trait-like and utilitarian decision-making styles, as well as risk-taking propensity, which are relatively poorly understood in heroin addiction.

Our data revealed that heroin-dependent individuals had a higher score on Procrastination but a lower score on Vigilance in the MDMQ compared with the HCs. These self-reported decision-making styles in HAs (i.e., less competent and more maladaptive) are similar to those of stimulant abusers in one prior study (31). In moral decision-making task, HAs displayed a utilitarian bias (i.e., made more utilitarian choices) in the self-involvement dilemmas, consistent with previous studies in polysubstance dependence and alcohol use disorders (32, 33, 39). Regarding risky decision-making, the BART is a typical task that assesses individuals’ propensity for risk-taking, with multiple regions underlying its neural bases in the reward network, salience network, and executive control network (60). Our study revealed that HAs exhibited an increased propensity for risk-taking, indexed by more averaged pumps and explosions on the BART, consistent with previous reports in treatment-seeking heroin abusers (25).

More important findings in this study were from the direct comparison between HAs and their siblings. Because the Siblings are free from any individual drug abuse, it is reasonable to speculate that commonalities in decision-making between HAs and Siblings may suggest a familial susceptibility (61). On trait-like decision-making styles (MDMQ), we found that both HAs and Siblings had a significantly higher score of Procrastination with a large effect size (Cohen’s *d* = 0.61-1.17) compared with HCs. On the risky decision-making task (BART), both HAs and Siblings also revealed an increased propensity for risk-taking (i.e., a greater adjusted number of pumps and more exploded balloons) than HCs, with a moderate to large effect size (Cohen’s *d* = 0.39-1.25). Logistic regression models further supported these findings, indicating that MDMQ Procrastination and BART average number of pumps were common factors positively predicting HAs and Siblings, which manifested a critical role of delaying decisions and risk-taking as the potential markers related to familial vulnerabilities for heroin use disorder. Specifically, previous family studies have shown that sibling pairs discordant for heroin dependence were correlated on BART risk-taking propensity (26), and impulsivity, sensation seeking, and novelty seeking are considered the vulnerability markers for stimulant drug addiction (35, 62). The measure of risk-taking by the BART has been associated with real-world risk behaviors and measures of sensation seeking, impulsivity, and deficiencies in behavioral constraint (49); therefore, our findings suggested that increased risk-taking propensities could be a potential familial susceptibility for heroin dependence. Nevertheless, it is worth noting that correlation analyses indicated a positive relationship between years of heroin use and the average number of pumps in the BART (*r*= 0.281, *p=*0.018), still pointing to the potential deteriorating impact of chronic heroin use on risk-taking behaviors (63). On the other part, Procrastination is a tendency to avoid making decisions by delaying decisions, and is considered a maladaptive decision-making style linked to the undesirable outcomes. Amphetamine-dependent individuals reported a greater tendency to delay decisions compared to healthy subjects (31), and Procrastination is a key predictor of lifetime psychostimulant use and current binge-eating behaviors (64, 65). In our study, the elevated traits of Procrastination in both HAs and Siblings might indicate a potential marker for heroin addiction. Due to the shared cognitive dysfunctions between HAs and their unaffected siblings (12), it is conceivable to speculate that the high levels of Procrastination may be related to deficits in cognitive control. Interestingly, impaired inhibitory control is emerged in both stimulant-dependent individuals and their unaffected siblings as a neurocognitive endophenotype for stimulant use disorder (43–45). Although our data do not allow a direct inference about its underlying causes, these findings revealed an important role of trait-like decision-making styles (Procrastination) implicated in the familial susceptibility for the development of heroin abuse and dependence.

Simultaneously, the specific effects of chronic heroin use on decision-making abilities were examined by the regression models comparing HAs and Siblings. A lower competent style (Vigilance) could predict the HAs when compared with Siblings (*OR* = 0.546, 95% *CI* = 0.414-0.722), while Siblings showed a comparable score of Vigilance with HCs. It seems that chronic heroin abuse might contribute to significant impairments in competent decision-making abilities (e.g., Vigilance), which is in line with previous reports among chronic stimulant abusers (31). Conversely, this finding may also indicate that an intact competent decision-making ability (Vigilance) could serve as an important protective factor against a history of drug use in the unaffected siblings. Furthermore, although the Siblings scored higher on Procrastination and exhibited a greater inclination towards risk-taking compared to HCs, they were not as “impaired” as the HA in these domains, which might be a result of being free from drug use. Further research is needed to uncover the bases of risk and protective traits identified in Siblings.

There are several limitations that should be noted in this study. Firstly, smoking status was not controlled, as is common practice in most addiction studies (61). However, we tested the possible effects of smoking behavior on our main results by including current smoking status as a covariate variable, but did not find any notable influence. Thus, the group differences in smoking may not change our findings significantly. Secondly, although the decision-making tasks have been widely used and validated in previous studies, all of them are hypothetical, especially the MDMT, and the results should be taken with caution. Future studies should examine actual decisions among the HAs with more ecologically valid tasks, such as that used in one previous study (66). Finally, several self-report scales were used in this study (e.g., MDMQ, SDS, SAS); thus, it is possible that a subjective bias may not have been completely avoided, and the findings in our study should be explained carefully.

Despite these limitations, this family study suggests that a maladaptive decision-making trait (i.e., Procrastination) and an elevated risk-taking propensity, observed in heroin-dependent individuals and their siblings, may serve as potential familial vulnerability markers implicated in heroin use disorder.

## Data Availability

The raw data supporting the conclusions of this article will be made available by the authors, without undue reservation.
