# Supplementary material for: Commonalities and differences in trait-like, risky, and utilitarian decision-making styles between abstinent heroin-dependent individuals and their siblings
Source: Front Psychiatry. 2026 Jan 8;16:1659008. doi: 10.3389/fpsyt.2025.1659008 (PMC12823923; doi:10.3389/fpsyt.2025.1659008)
Supplement: Supplementary file 1 [file Table1.doc]

Supplementary Materials

**Table S1** Variance inflation factor (VIF) for the variables in the logistic regression models.

| Models | Variance inflation factor (VIF) in the models | | |
| --- | --- | --- | --- |
|  | Model 1: HAs (1) *vs.* HCs (0) (*n*=144) | Model 2: Siblings (1) *vs.* HCs (0) (*n*=143) | Model 3: HAs (1) *vs.*Siblings (0) (*n*=139) |
| **Step 1** |  |  |  |
| Current smokers (yes=1, no=0) | 1.246 | 1.100 | 1.054 |
| SAS scores | 1.460 | 1.057 | 1.225 |
| SDS scores | 1.521 | 1.127 | 1.317 |
| **Step 2** |  |  |  |
| MDMQ Vigilance | 1.458 | 1.075 | 1.404 |
| MDMQ Buck-passing | 2.732 | 1.860 | 2.366 |
| MDMQ Procrastination | 2.239 | 1.742 | 1.782 |
| MDMQ Hypervigilance | 2.535 | 1.713 | 2.266 |
| BART average number of pumps | 1.812 | 1.216 | 1.295 |
| BART total number of explosions | 2.002 | 1.210 | 1.259 |
| MDMT ratio of utilitarian choices ^a^ | 1.211 | 1.259 | 1.283 |
| MDMT ratio of utilitarian choices ^b^ | 1.196 | 1.361 | 1.372 |

^a^ Self-involved scenarios. ^b^ Other-involved scenarios. HAs, Heroin-dependent individuals; HCs, Healthy subjects.

SAS, the Self-Rating Anxiety Scale. SDS, the Self-rating Depression Scale.

MDMQ, the Melbourne Decision-Making Questionnaire. BART, the Balloon Analogue Risk Task.

MDMT, the Moral Decision-Making Task.

**Table S2** Pearson correlations between main task scores and SAS and SDS scores (r, n=213)

| Task scores | General intelligence # | SAS scores | SDS scores |
| --- | --- | --- | --- |
| MDMQ Vigilance | -0.085 (*p*=0.219) | 0.018 (*p*=0.792) | -0.064 (*p*=0.351) |
| MDMQ Buck-passing | -0.002 (*p*=0.978) | 0.016 (*p*=0.814) | -0.045 (*p*=0.518) |
| MDMQ Procrastination | -0.123 (*p*=0.074) | 0.085 (*p*=0.219) | -0.021 (*p*=0.765) |
| MDMQ Hypervigilance | -0.134 (*p*=0.053) | 0.117 (*p*=0.088) | -0.069 (*p*=0.315) |
| BART average number of pumps | -0.139 (*p*=0.051) | 0.052 (*p*=0.452) | 0.132 (*p*=0.054) |
| BART total number of explosions | -0.132 (*p*=0.054) | 0.025 (*p*=0.718) | 0.072 (*p*=0.297) |
| MDMT ratio of utilitarian choices ^a^ | -0.058 (*p*=0.397) | 0.122 (*p*=0.074) | -0.025 (*p*=0.714) |
| MDMT ratio of utilitarian choices ^b^ | -0.103 (*p*=0.132) | 0.089 (*p*=0.194) | -0.004 (*p*=0.955) |

# Estimated by the intelligence quotient (IQ) scores on the Raven’s Standard Progressive Matrices (RSPM).

^a^ Self-involved scenarios. ^b^ Other-involved scenarios.

SAS, the Self-Rating Anxiety Scale. SDS, the Self-rating Depression Scale.

MDMQ, the Melbourne Decision-Making Questionnaire. BART, the Balloon Analogue Risk Task.

MDMT, the Moral Decision-Making Task.
